# Overstepping the upper refractive index limit to form ultra-narrow photonic nanojets

**DOI:** 10.1038/s41598-017-05781-4

**Published:** 2017-07-17

**Authors:** Guoqiang Gu, Jun Song, Hongda Liang, Mengjie Zhao, Yue Chen, Junle Qu

**Affiliations:** Key Laboratory of Optoelectronic Devices and Systems of Ministry of Education and Guangdong Province, College of Optoelectronic Engineering, Shenzhen University, Shenzhen, 518060 China

## Abstract

In general, photonic nanojets (PNJs) occur only when the refractive index (*Ri*) difference between the microparticle and background media is less than 2. The minimum full width at half-maximum (FWHM) of the PNJ is ~130 nm (approximately one-third of the illumination wavelength *λ* = 400 nm) formed within the evanescent field region. This paper proposes and studies a method to overstep the *Ri* upper bound and generate ultra-narrow PNJs. Finite element method based numerical investigations and ray-optics theoretical analyses have realized ultra-narrow PNJs with FWHM as small as 114.7 nm (0.287 *λ*) obtained from an edge-cut, length-reduced and parabolic-profiled microparticle with *Ri* = 2.5 beyond evanescent decay length. Using simple strain or compression operations, sub-diffraction-limited PNJs can be flexibly tuned on the order of several wavelengths. Such ultra-narrow PNJs offer great prospects for optical nonlinearity enhancements of greater enhancing effect, optical nanoscopy of higher spatial resolution, optical microprobes of smaller measurement accuracy, nano/micro-sized sample detections of higher sensing sensitivity, nanoscale objects of more accurate control, advanced manufactures of smaller processing size, optical-disk storage of larger data capacity and all-optical switching of lower energy consumption.

## Introduction

Tightly focusing light onto the shadow-side surface of a plane-wave-illuminated dielectric microparticle has opened the interesting field photonic nanojets (PNJs)^1^. Such a nonresonant, high-intensity light beam can propagate along a path extending beyond the evanescent field region without significant diffraction, and maintain a subwavelength full width at half-maximum (FWHM) transverse beamwidth. Over the past decade, many PNJ aspects have been extensively studied, such as backscattering enhancement^2^, Raman scattering^3^, PNJ induced optical force^4^, coupled resonator optical waveguide^5^, ultrahigh density optical data storage^6^, optical trap assisted nanopatterning^7^, super-resolution optical imaging^8^, ultrafast all-optical switching^9^, and manipulation and detection of nanoparticles and cells^10^. PNJ formation characteristics are determined by the considered microparticles’ material properties and geometrical morphologies, exterior environments, and illumination wavelength scales. Setting and transforming these parameters in combination with structural composition design means that, the PNJ attribute parameters can be reasonably controlled. Non-evanescent propagated PNJs with narrower FWHM and higher field intensity provide increased force, transport, nonlinearity and super-resolution effect^11^.

Many papers have studied improving and optimizing these properties to further explore and efficiently utilize PNJs. When the refractive index (*Ri*) of the microparticles are increased to a certain value (*Ri* ~ 1.75 in Fig. S1a), the tightly focused beam forms a PNJ that moves in the opposite direction of the illumination light towards the shadow-side surface of the microparticle, and FWHM (*ω*) narrows (from 0.43 to 0.39 *λ*). In principle, larger *Ri* produces smaller *ω* ^1^. However, the light refraction between the microparticle and background media, the nominal ‘ultra-narrow PNJ’ to first be close to the microparticle surface then gradually become embedded and fully confined within the microparticle when their refractive indices differ by greater than approximately 2 (*Ri* = 2, 2.25, 2.5 in Fig. S1a). The reported minimum *ω* is ~130 nm with illumination wavelength *λ* = 400 nm, the focal point is located almost at the microsphere surface boundary, and the microsphere is small, ~1 *μ*m^2^. This implies that there is an objective trade-off between further narrowing FWHM beamwidth below the classical diffraction limit and retaining PNJ outside the microparticle surface, which cannot be overcome by simply introducing a dielectric microparticle with higher *Ri*. Lengthening PNJs towards regions farther away from the outside surface of the microparticle could be realized by replacing the homogeneous dielectric microparticle with several concentric shells of graded refractive indices or immersing in a liquid environment^12, 13^. However, this longer PNJ is produced only with the great sacrifice of widening the transverse beamwidth (e.g. ~2.5 *λ* in ref. 13).

**Figure 1:**
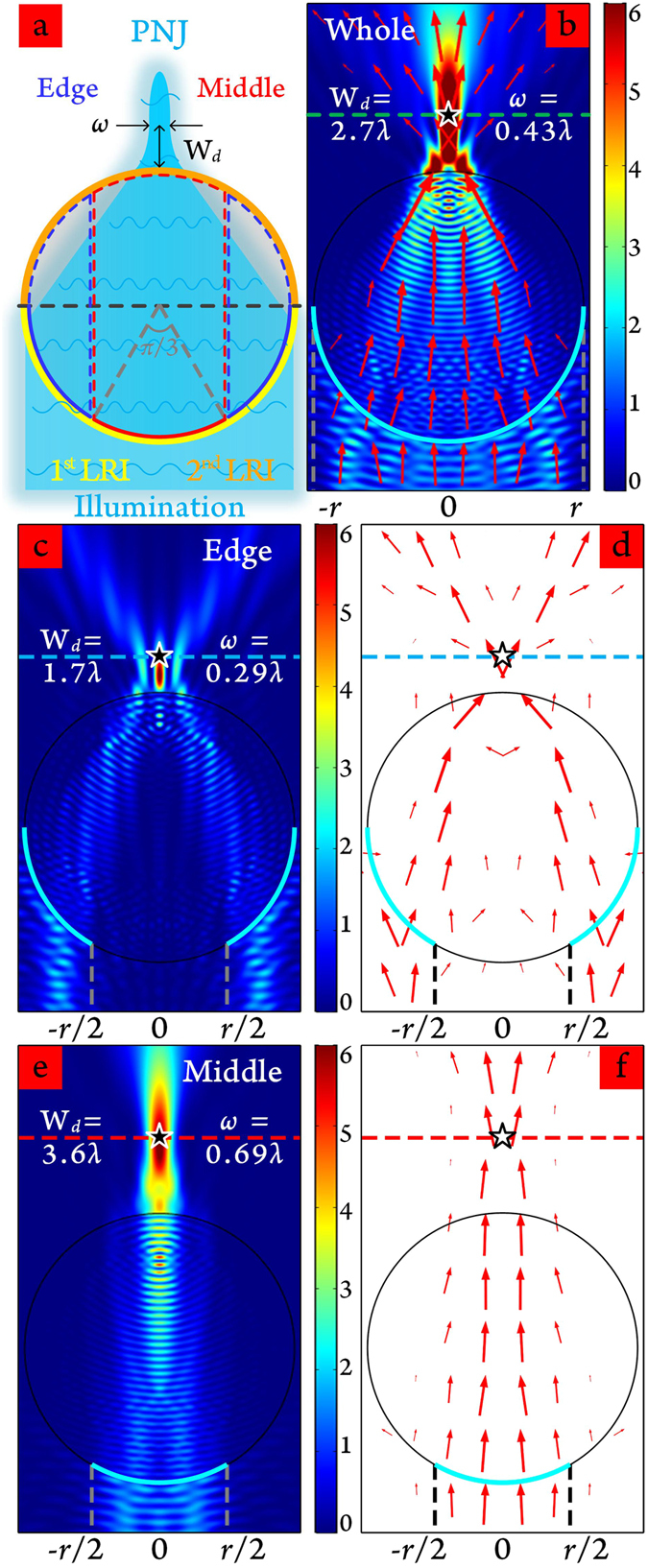
(**a**) Structural components of cylindrical microparticles for generating PNJ under light illumination, including the 1^*st*^ (yellow semi-circular arc) and the 2^*nd*^ (orange semi-circular arc) LRIs. Three regions with the same arc lengths of the 1^*st*^ LRI: one middle region (red arc) and two edge regions (blue arcs). W_*d*_ is the distance from the surface of the microparticle to the position of maximum light intensity, *ω* is the minimum FWHM beam width. (**b**) Electric field intensity of plane wave illuminating the whole boundary of the 1^*st*^ LRI. Wavelength *λ* = 400 nm, microparticle radius *r* = 2.5 *μ*m and the arrows denote the distribution of Poynting vector field. (**c**) Edge regions and (**e**) middle region are illuminated in the vertical direction, forming PNJs. (**d**,**f**) the corresponding Poynting vector distributions of the irradiation areas located at the edge and middle regions, respectively. The five-pointed stars in (**b**)–(**f**) mark the positions of maximum light intensity. The *Ri* of the microparticle is 1.5.

Morphology changes can modify spatial features and field distributions of PNJs in different degrees. W.M. Saj investigated a two-dimensional (2D) structure of isosceles triangle shaped metal-insulator-metal waveguide stack illuminated by H-polarized light beam to obtain a PNJ beyond the sharp edge with focus width concentrating to 184 nm (0.368 times the illumination wavelength of 500 nm)^14^. Kotlyar *et al*. designed square-profile microsteps on a silica substrate to produce PNJs with their intensity six times higher than the incident light and their FWHM as small as 247 nm (0.39 times the illumination wavelength of 633 nm) at the case of square side and height equalling 0.6 *μ*m and 500 nm, respectively^15^. Geints *et al*. had systematically summarized the modification of incident optical radiation and control of PNJ property by shaped wavelength-sized particles in their recent publication^16^. By varying the spatial shape of the micrometer scatters into non-spherical, non-symmetrical and composite structures, the key parameters of PNJs (length, width and light intensity) can be efficiently improved. Other changes in shape including core and hemispherical shell configurations^17, 18^, liquid-filled hollow microcylinders^19^, and more exotic structures, such as the binary spiral axicon and corrugated cylinder^20, 21^, are the other ways to manipulate the morphology of the PNJs. However, since the formed PNJs are initially restricted to the *Ri* upper bound, a slight outward extension will inevitably cause the PNJ converge more slowly, leading to increased FWHM, above the record of ~130 nm. For almost all the morphology changes, the complete entities or partial engineered structures are focused on transforming the second light refraction interface (LRI, the orange semi-circular arc in Fig. 1a), whereas the shape boundaries inducing the first light refraction (the yellow semi-circular arc part in Fig. 1a) are always used integrally. Very few studies have investigated influences on PNJ formation from the breaking or altering the integrity of the first LRI. Yan *et al*. lightly discussed the possible function of a pupil mask introduced to shield a portion of the illumination light reaching the microsphere surface^22^. The minimum FWHM (185.23 nm) was somewhat less than 200 nm, while the obtained PNJ via the convergence of unshielded light should pass through the microlens with lower *Ri* (1.46). Wang *et al*. compared image contrast and analysed the imaging mechanism of different illumination conditions for super-resolution imaging with near-field assisted white light interferometry^23^. Tight focus was produced by illuminating the boundary area. However, the focused spot, was confined within the evanescent field region and the *Ri* difference of the BaTiO_3_ microsphere and water media was only ~1.43.

The first LRI can be divided into two distinct parts constituted by two edge regions and one middle region (Fig. 1a). When light illuminates the whole boundary of the first LRI, the usual case shown in Fig. 1b, the working distance of the generated PNJ (W_*d*_ ≈ ~2.7 *λ*) is greater than the evanescent decay length (~*λ*/2*π*)^24^, and FWHM *ω* = 0.43 *λ* is smaller than the diffraction limit (0.5 *λ*). Time-averaged Poynting vectors (red arrows) show two convergences on the first and second LRIs between air and the cylindrical microparticle followed by an outward scattering. If the irradiation area is set at the two edge regions, a PNJ of shorter working distance (1.7 *λ*) and smaller FWHM (*ω* = 0.29 *λ*) appears at the rear surface of the microparticle (Fig. 1c). The Poynting vectors’ distribution indicates that incident light is initially refracted into the microparticle with large amplitude and later rapidly gathered and diffused after exiting from the second LRI (Fig. 1d). When only the middle part of the microparticle is irradiated, since the incidence angles are relatively small (≤30°), the light beams travel through the first and second LRIs with very little deflection (Fig. 1e,f). The PNJ forms containing the longest W_*d*_ (3.6 *λ*) and largest *ω* (0.69 *λ*) relative to the usual and edge cases. For these three cases as well as the other morphology changes, the starting points of the emergent rays are situated in the centre region of the second LRI. As the particle size increases, the PNJ moves farther away (Fig. S1b). Following the structure discussed previously, the current paper proposes a method to preserve the microparticle’s middle region and chip off the edge regions, together with appropriate optimal design can realize microparticle superlenses with overstepped upper *Ri* limits and generate ultra-narrow PNJs. PNJs formed from high refractive index (*Ri* = 2.5) microparticles have been successfully demonstrated. Finite element method (FEM) based numerical simulation and ray optics based theoretical analysis showed that parabolic-profiled microparticles could well produce PNJs with ultra-narrow beam width (as small as 114.7 nm) located at a definite space outside the evanescent field region. By simply exerting mechanical strain or compression, sub-diffraction-limited PNJs can be flexibly tuned within several wavelengths.

## Results and Discussion

**Figure 2:**
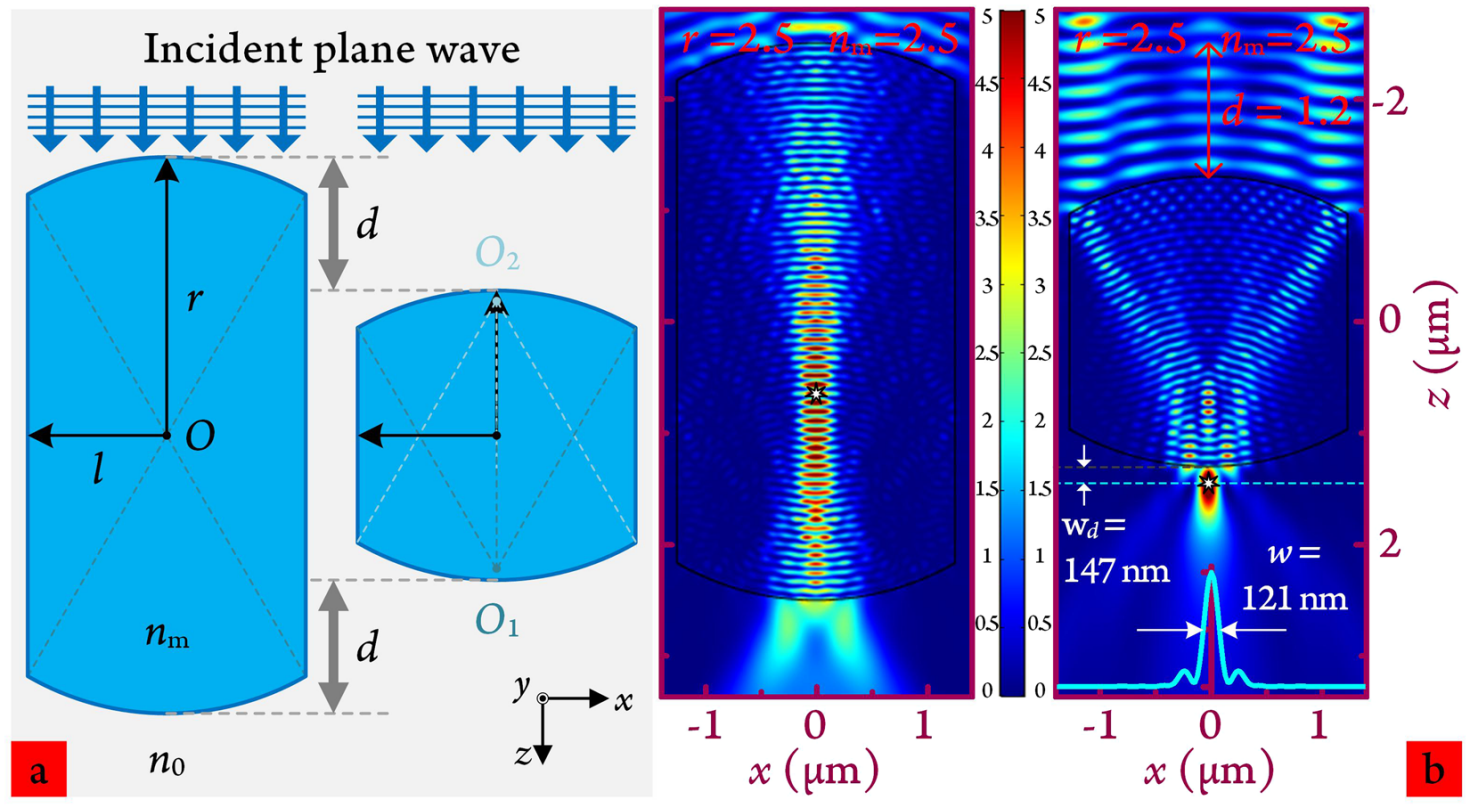
(**a**) Schematic illustration of MPCM transforming to R-MPCM by reducing the structure length in the vertical direction. The incident plane wave was propagated from top to bottom. (**b**) 2D electric field distributions of MPCM and R-MPCM (with reduced length 1.2 *μ*m) obtained from FEM-based full wave simulation. Inset: transverse intensity profile at the focal point. The two seven-pointed stars indicate maximum light intensity of the electric field intensity distribution.

Consider the electromagnetic interaction between the selected middle part of the cylindrical microparticle(MPCM), and an unpolarised monochromatic plane wave incident along the central symmetrical axis. The materials of the infinite MPCM and its surrounding medium are assumed to be homogeneous, isotropic and lossless. Figure 2a, left panel, shows the early-evolved MPCM model, where *O* is the central point of the structure; *r* and *l* represent the distances from *O* to MPCM’s uncut and cut sides, respectively; and *n*_*m*_ and *n*_0_ are the refractive indices of MPCM and surrounding medium, respectively. The two darkened intersection points, *O*_1_ and *O*_2_, are the centres of the top and bottom arcs respectively.

Since *Ri* is increased to *n*_*m*_ = 2.5, the highly concentrated optical field remains confined within the particle even when using the MPCM structure. Figure 2b, left panel, shows the electric field distribution for an MPCM with *r* = 2.5 *μ*m and *l* = *r*/2 = 1.25 *μ*m. The maximum light intensity (Fig. 2b, asterisk) is near to the MPCM central point. This result can be easily understood using the MPCM energy flow plot(see Fig. S2a, Supporting Information). Tracing the energy flow, there is a long term optical field distribution that is almost parallel to the lower half part of MPCM along the direction of light propagation. It follows, then, that such a strongly enhanced field could shifted into the external space for forming PNJ by reducing the structure length. Figure 2a, right panes, shows the reduced-MPCM (R-MPCM). To simplify the case, without loss of generality, we reduce *d* along the *z* axis for both sides without changing the shape of the whole structure and the *x* axis dimension. As shown in Fig. 2b, right panel, a real PNJ is formed at W_*d*_ = 147 nm with *d* = 1.2 *μ*m. Due to the adoption of high-*Ri* material, FWHM is significantly narrowed to less than the previously reported minimum *ω* = 130 nm, and far lower than Abbe diffraction limit. The electric field profile of the maximum light intensity along *x* axis has minimum *ω* = 121 nm. The energy flow streamlines, as shown in Fig. S2b, represented the out-shift rule and trend. The convergence point falls on the outside of the particle with decreased MPCM longitudinal length. The same studies were performed for MPCM and R-MPCM of *Ri* = 2.25, as shown in Fig. S3a,b. Since the FWHM beam for portion gathered optical field in case of MPCM (*ω* = 193 nm) and fully formed PNJ in case of R-MPCM (*ω* = 157 nm) are both higher than the aforesaid value of 130 nm (which is not the aim of this work), the related properties of these two cases will not be continuously researched in the following.

**Figure 3:**
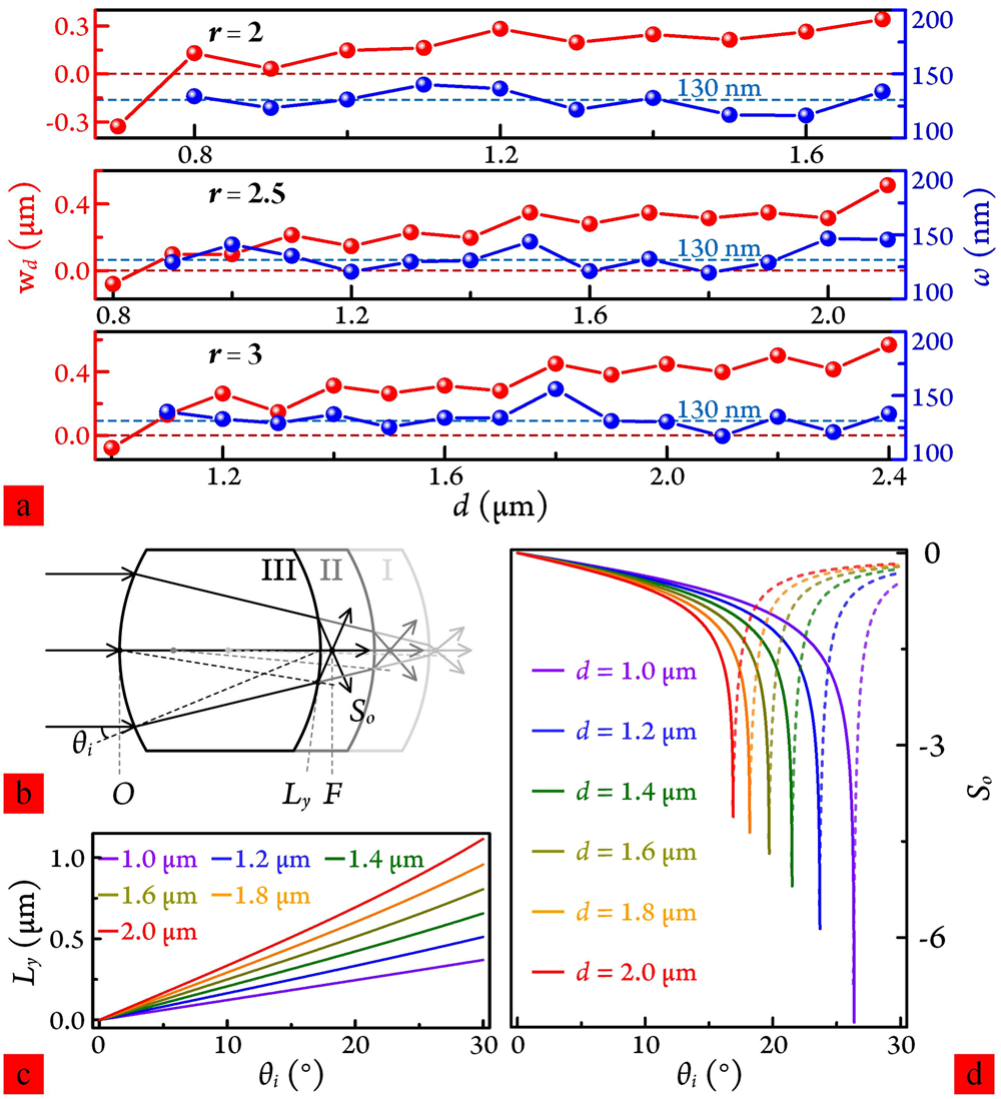
(**a**) Working distance W_*d*_ and FWHM beam width, *ω* of PNJs for *r* = 2, 2.5 and 3 *μ*m as a function of reducing length *d*. (**b**) Ray tracing analysis for plane-wave-illuminated R-MPCM for the three cases of (**a**). (**c**) Emitting point locations, and (**d**) emergent ray slopes for *r* = 2.5 *μ*m and length reduction from *d* = 1.0 to 2.0 *μ*m, respectively.

To investigate behavioural details, we studied the impact of different reducing lengths on PNJ out-shifting and shaping for different R-MPCM dimensions. Figure 3a shows the simulation results for *Ri* = 2.5 and *r* = 2, 2.5 and 3 *μ*m. Length reduction was increased with step size = 0.1 *μ*m. In general, when the reducing length is smaller than a specific value, propagation light is focused within the particle. No PNJ was produced, see the red and blue symbol-line graphs, the corresponding W_*d*_ is expressed as a negative and the half-width of the focusing spot was not considered. As the reducing length increases, although the W_*d*_ moves forward and backward to some extent, the variation curve as a whole has an ascending trend. These fluctuations occurred almost simultaneously for FWHM, while *ω* for all three cases (given in the figure) is significantly below the diffraction limit. Maximum W_*d*_ ≈ 1.42 *λ* (for case *r* = 3 *μ*m) and minimum *ω* was approximately 117.5 nm (for case *r* = 2 *μ*m). This is consistent with previous analyses on the relationship between the microparticle curvature radius and PNJ distribution characteristics. There are also several discrete intervals where *ω* < 130 nm. The discontinuous changes can be qualitatively analysed and interpreted using the classical ray optics approach.

Figure 3b shows the geometrical optics model, tracing rays through the R-MPCM for different reducing lengths. However, given the main focus of the current paper, the small number of reflected light reaching the second LRI was not followed-up or analysed. Three cases: I, II and III, response to the light trajectories are listed and discussed. From Snell’s law, plane wave refraction occurs at a boundary between the microparticle and surrounding air. *O* indicates the centre of the second LRI, *θ*_*i*_ is the incidence angle, *F* denotes the focal point of the resulting PNJ, and *L*_*y*_ and *S*_*o*_ are the ordinate values of emitting points and the slopes of the emergent rays, respectively. The position change of the focal point can be clearly seen from the figure. Interestingly, it is not a monotonically increasing function with respect to reducing length from Case I to Case III. Regarding R-MPCM for *r* = 2.5 *μ*m, the location distributions (*L*_*y*_) and the slope curves (*S*_*o*_) with different length reductions (*d*) are shown in Fig. 3c,d. To more clearly display *L*_*y*_ and *S*_*o*_ differences, curves with length reduction step 0.2 *μ*m are shown from *d* = 1.0 to 2.0 *μ*m. For the case step length = 0.1 *μ*m, the curves show the same change trend as Fig. 3c,d. Since the considered structure is axially symmetric, the positive value part of the incident angle *θ*_*i*_, 0–30° was selected to illustrate the results. When *d* is small (Case I), *L*_*y*_ positions of emitting points confined within a very small area (Fig. 3c, violet solid line). Only a fraction of light beams close to two cut sides, as shown in Fig. 3d, fairly rapidly converge and diverge. The variations of the slopes (*S*_*o*_) for most of the emergent light beams are moderately slow. For this case, the first factor has a dominant role in generating a PNJ of short working distance and relatively narrow beam width. When *d* is increased (Case II), as an example of *d* = 1.2 *μ*m, the slopes of most emergent rays are overall higher than the values for Case I, and the optical fields naturally focus to a tighter spot. However, since the emitting points distribute over a wider area and the slopes for more light beams close to the edge are smaller than for Case I, emergent rays converge to somewhere far away. As reducing length is further increased, the influence originating from a growing number of optical fields that gradually move to the middle part of the microparticle will become more. Three factors commonly affect PNJ formation. Firstly, wider distribution of emitting points causes further intersections of emergent rays with *z* axis for the beams of having the same slopes relative to the case of smaller reducing length. Secondly, faster convergence and divergence speed of the light rays produces smaller FWHM. Thirdly, as discussed at the beginning of this paragraph, the more optical fields of relevance to large incident angles are lower in slopes, the light focusing of wider beam width and longer working distance forms. The joint action of these three factors cause the PNJs to show forward and backward movements and size fluctuations with incrementing length reduction. From a larger perspective, an expanding distribution area of *L*_*y*_ and a flatting slope curve of *S*_*o*_ generally cause W_*d*_ to extend outward.

**Figure 4:**
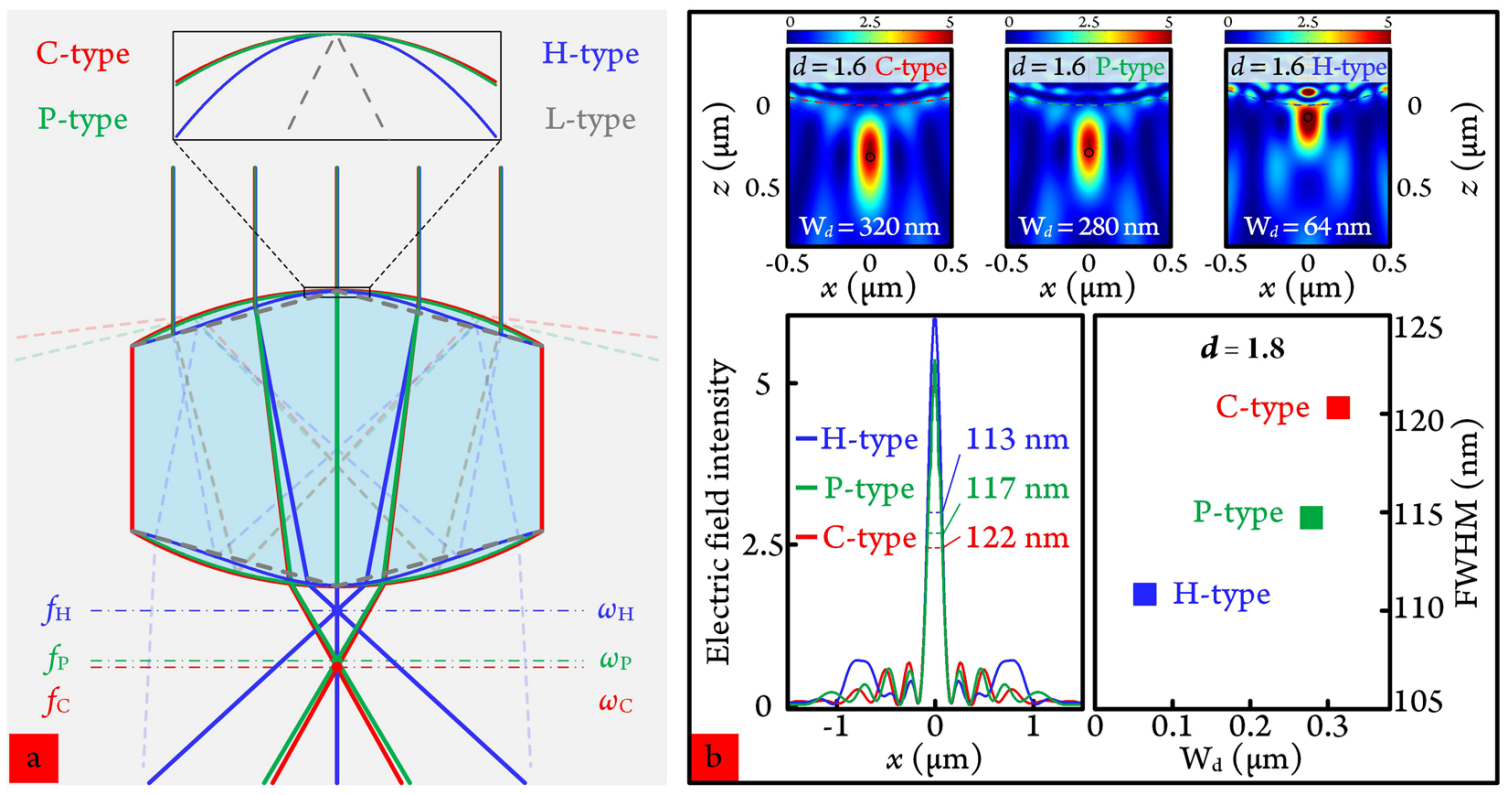
(**a**) Four boundary profiles for narrowing the transverse beam widths of the PNJs. C-type: circular type, P-type: parabolic type, H-type: harmonic-oscillating type, L-type: linear type. (**b**) FWHM beam widths for C-, P- and H-type R-MPCMs with (left panel) *d* = 1.6 *μ*m and (Right panel) *d* = 1.8 *μ*m. Insets at the top: the electric field distributions of C-, P- and H-type R-MPCMs for *d* = 1.6 *μ*m.

R-MPCMs, including those discussed previously, are all microparticles with circular boundaries. From the relation between PNJ attributive characteristics and microparticle boundary curvatures, PNJ can be further modulated by changing the microparticle boundary profiles, as show in Fig. 4a for four shape profiles: C-type (circular), P-type (parabolic), H-type (Harmonic-oscillating^25^) and L-type (linear). The inset at the top of Fig. 4a magnified profile differences for these four types. The curvatures for C- and P-type boundary profiles are very similar, while the H-type R-MPCM is visibly higher than the previous two. The curvature of L-type R-MPCM is zero on two symmetric boundaries. Because of the total internal reflection (TIR) easily occurring around the boundary for L-type microparticles and the considering parallelism of the two opposite sides of this structure (see the supporting information in Fig. S4a), it is just given as a lower bound line on boundary profile transformation and no additional discussion is added to such R-MPCM. *f*_*C*_, *f*_*P*_ and *f*_*H*_; *ω*_*C*_, *ω*_*P*_, *ω*_*H*_ are respectively the focal points and beam widths of the generated PNJs from these three profiled R-MPCMs. Choice of P-type and H-type profiles, as optimization options, is mainly due to the consideration of developing three-dimensional (3D) high-*Ri* superlens with controllable heating-and-deforming or self-assembling methods in the future^25–27^.

Figure 4b compares the transverse and longitudinal electric field distributions of C-, P- and H-type R-MPCMs for the cases of *d* = 1.6 and 1.8 *μ*m. The selection of these reducing lengths are that *ω* is small and the working distances are comparatively long. Since the curvature of P-type R-MPCM is a little larger than the R-MPCM of C-type, the *ω* is slightly narrowed from 122 to 117 nm and W_*d*_ is decreased from 320 to 280 nm. For *d* = 1.8 *μ*m, *ω* and W_*d*_ have the same trend, 120 to 114.7 nm and 315 to 280 nm, respectively. For H-type R-MPCMs, though *ω* has decreased to a new level of as small as 113 nm and 110.8 nm for these two cases, the focal point positions (64 nm for both cases) return to the area approaching the evanescent field (~*λ*/2*π* ≈ 63.7 nm). Figure S4b–g shows ray tracing and energy flow closely demonstrated this change rule.

Several possible methods can be used to manufacture such new type microparticles. From the 2D point of view, low divergence PNJs obtained from Si_3_N_4_ microdisk (*Ri* = 2.1) offers a good reference and solution^28^. The infinitely long P-type R-MPCMs may be fabricated through E-beam lithography and inductively coupled plasma etching techniques with potentially available materials of *Ri* > 2, such as transparent ceramic, diamond, silicon, etc. When it extends to the 3D structures, chalcogenide glasses with high linear refractive index (2–3), broad transparency (visible to mid-infrared) and low to moderate glass transition temperature (*T*_*g*_ ≈ 100–400 °C) are good candidate materials for the preparation^29, 30^. By using the aforementioned heating-and-pulling or softening-and-compressing methods^25, 26^, the chalcogenide glass optical fibers will be made into 3D H- or P-profiled microparticles.

**Figure 5:**
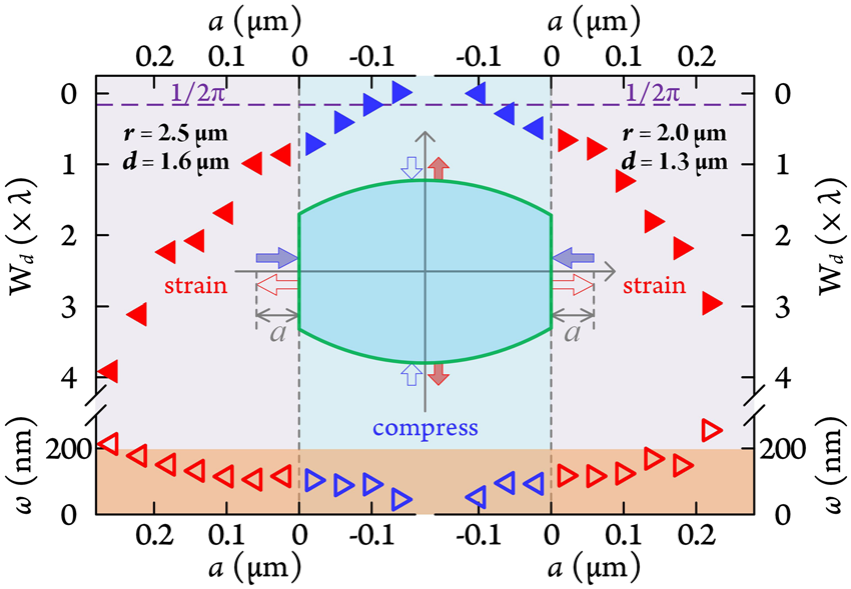
(**a**) Tuning PNJ parameters (working distance, W_*d*_, and FWHM beam width *ω*) by mechanically straining or compressing the P-type R-MPCMs for *r* = 2.5 *μ*m, *d* = 1.6 *μ*m; and *r* = 2.0 *μ*m, *d* = 1.3 *μ*m. Violet dashed line indicates the decay length of evanescent wave (*λ*/2*π*). Inset: schematic illustrating strain and compression actions.

Manipulating and controlling PNJ characteristics to adapt to allow parallel detection with microlens arrays, deep nano-structure exploration within biological objects, scanning rough samples through microparticle assisted optical microscopy and controlling switching energies in all-optical applications are very important. PNJs with smaller FWHM beam width could well enlarge the tuning range under the diffraction limit. Figure 5 shows numerical strain and compression tuning of two P-type R-MPCMs, with *r* = 2.5 *μ*m, *d* = 1.6 *μ*m and *r* = 2.0 *μ*m, *d* = 1.3 *μ*m, for *ω* and W_*d*_ relying the stretched or compressed length (*a*) along the direction perpendicular to the two cut sides. Force is exerted onto the walls of the cut sides and induces a change in horizontal dimension, longitudinal dimension and boundary curvature of the R-MPCM. We assumed that the shape profile and length of the cut sides remain constant during deformation. When the walls are pulled outwards (*a* > 0), microparticle longitudinal length and curvature radius decrease simultaneously, accordingly the W_*d*_ and *ω* of the PNJ are increased. In contrast, under compression (*a* < 0), W_*d*_ and *ω* are decreased due to increases of longitudinal dimension and boundary curvature. This is consistent with changing PNJ characteristics by transforming particle length and surface topography. The focal spot size of high-*Ri* microparticles mean PNJs become narrower, and the sub-diffraction-limit FWHM beam width can be tuned to sub-100 nm (87.6 nm on the microparticle surface for *r* = 2.0 *μ*m, *d* = 1.3 *μ*m) considering light focusing within the evanescent wave. The tunable region of the focal point positions ranges from 0 to ~3 *λ* for these two cases. Thus, PNJ parameters are fairly sensitive to even one-dimensional size adjustment, which a good basis for using the variation of PNJ parameters to sense the applied force.

## Conclusions

In summary, the PNJ phenomenon, in the past, can only appear at the *Ri* difference of less than 2 between the microparticle and background media. The FWHM beam width is accordingly limited to a certain value of ~130 nm and the main body of this minimum PNJ is embedded within the microparticle. Traditional methods for improving and optimizing PNJ attribute parameters (e.g. beam width and working distance) are mostly focused on changing the dielectric properties, geometrical morphologies and surrounding media of the microparticles involving integrate shape boundaries. Very few investigations have been explored the influences of the break or alteration of the integrities of the first LRI to the PNJ formation. Based on the comparison and analysis of the utilization of diverse optical field distributions formed from interactions between light and distinct microparticle parts of the first LRI of a cylindrical microparticle, a method of overstepping upper *Ri* limits and realizing ultra-narrow PNJs has been proposed in this work. Using the middle part of the considered microparticle, MPCM, the objective trade-off between reducing FWHM with high-*Ri* materials, which leading to inward PNJ movements, can be remedied. Real PNJs with beam width lower than the reported minimum (~130 nm) were obtained from longitudinal length reduced MPCMs. Further transforming the R-MPCM outer profile into P-type, the PNJ FWHM beam widths are decreased to ultra-narrow values of 117 nm for *Ri* = 2.5, *r* = 2.5 *μ*m, *d* = 1.6 *μ*m; and 114.7 nm for *Ri* = 2.5, *r* = 2.5 *μ*m, *d* = 1.8 *μ*m. FEM-based numerical results and ray trace analyses jointly verified the proposed process. Two simple mechanical actions, strain or compression, applied to optimally designed microparticles make the sub-diffraction-limit PNJs flexibly tunable from the evanescent field region to as far as 3 *λ*.

PNJs generating from microparticles of higher *Ri* and containing ultra-narrow FWHM beam widths are likely to show huge prospects and particularly important roles in a broad range of areas: such as the stronger enhancement effect and lower background noise in fields of enhancing the backscattering, fluorescence, Raman scattering and other non-linear optical signals; the higher spatial resolution for the techniques of white-light nanoscope, confocal microscope, photoacoustic imaging, stimulated Raman scattering imaging, coherent anti-Stokes Raman spectroscopic imaging and other ultramicroscopic techniques; the tinier optical microprobes for precision surgery in the eye and brain, noninvasive diagnosis of specific molecules in body fluids, careful analysis of internal composition within biological cells and other probing tools in biology/medicine; the higher sensing sensitivity in detecting metal/dielectric nanoparticles, proteins, viruses, cell fragments and other biological/chemical molecules; the more accurate control for trapping and manipulating nanoparticles, viruses, cells, molecules and other nanoscale objects; the finer processing precision for microparticle assisted laser nano-fabrication and optical trap-assisted nanopatterning and nanolithography; the larger information recording for optical data storage devices; the lower energy consumption for PNJs and semiconductor nanoparticles integrated all-optical switching; and the simpler alternative to replace conventional complex microscope objectives. We believe that such PNJs are potentially the more powerful tools for a wide variety of related applications in photonics, biology, chemistry, medicine and material sciences.

## Methods

An FEM-based simulation using Maxwell’s equations was performed, in this paper, to study the internal and external field distributions of the two-dimensional (2D) MPCM. All the simulation results are obtained through the COMSOL Multiphysics commercial software package. The distances from the central point of the structure to the uncut and cut sides of MPCM, *r* and *l*, and the reducing length of *d* are expressed using the unit of *μ*m. All the investigated microparticles are surrounded by air medium. Plane waves with fixed wavelength of *λ* = 400 nm and unit intensity along positive *z* direction incident onto the side surface of MPCM. The perfectly matched layer absorbing boundary condition is used to fully absorb the outward waves and eliminate undesired back reflections. Non-uniform meshes with *Ri*-dependent element size (8 nm for MPCM) are fine enough for the computational accuracy.

## Electronic supplementary material

Supporting Information
