# Supplementary material for: Overstepping the upper refractive index limit to form ultra-narrow photonic nanojets
Source: Sci Rep. 2017 Jul 17;7:5635. doi: 10.1038/s41598-017-05781-4 (PMC5514067; doi:10.1038/s41598-017-05781-4)
Supplement: Supplementary file 1 — Supporting Information [file 41598_2017_5781_MOESM1_ESM.pdf]

SUPPORTING INFORMATION

Overstepping the upper refractive index limit to form ultra-narrow photonic nanojets

Guoqiang Gu, Jun Song*, Hongda Liang, Mengjie Zhao, Yue Chen and Junle Qu*

Key Laboratory of Optoelectronic Devices and Systems of Ministry of Education and Guangdong Province, College of Optoelectronic Engineering, Shenzhen University, Shenzhen 518060, China

*songjun@szu.edu.cn & jlqu@szu.edu.cn

1. Influence of microparticle refractive index and size

The High-resolution finite element method (FEM) computational electromagnetic model is a reliable technique to investigate spatial field distributions of plane-wave-illuminated two-dimensional (2D) cylinders or three-dimensional spheres. The numerical study of light propagating through a homogeneous, isotropic, lossless, and infinitely long microcylinder and surrounding air media was implemented by solving Maxwell's equation using the COMSOL Multiphysics commercial software package.

First, we considered a 2D transverse electric wave incoming along the axis of a microcylinder of fixed radius (r = 2.5 μm). Light with optical wavelength λ = 400 nm propagated from bottom to top to irradiate the bottom semicircle of the particle. Figure S1a shows the evolution of photonic nanojets (PNJs) with respect to the refractive index (*Ri*) increasing from 1.5-2.5. Although the full width at half-maximum (FWHM) transverse beam width (ω) of the focusing light were dramatically decreased from 0.43 to 0.21 λ, the real PNJ disappeared when the refractive indices exceeded the upper bound $Ri \approx 2$. The five-pointed stars in Fig. S1a show the convergence points gradually approached to the rear side surface until fully entering within the microcylinder. The variation trend was fitted with a nonlinear decreasing curve (Fig. S1a, solid orange line). The efficient working distance (W_d) only existed where $Ri \leqslant$ upper *Ri* limit.

In contrast, for microparticles with curved surfaces, the working distance and beam width of the PNJs are increasing with decreasing curvature radius[1]. We conducted a similar numerical investigation on a 2D microcylinder, illuminating its selected surface boundary, i.e., the middle part of the cylinder. The refractive index was Ri = 1.5, which ensure PNJs formed outside of the microparticle surface. Figure S1b shows that W_d and ω increase with increasing of particle radius from r = 2 to 3 μm. All the focusing beams were real PNJs. A nonlinear increasing curve (Fig. S1a, solid orange line) fitted the PNJ focal points (Fig. S1a, five-pointed stars) well.

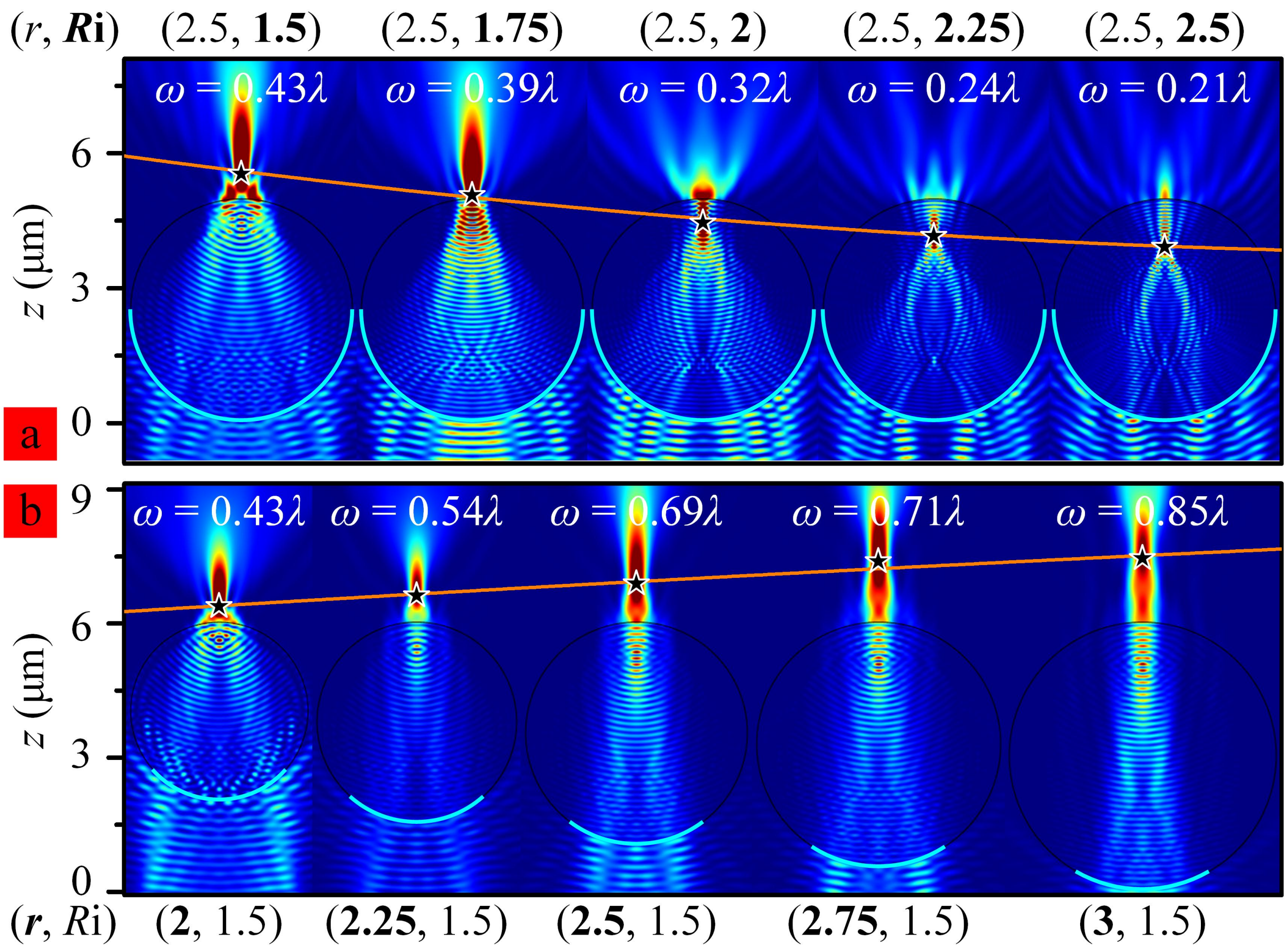

Figure S1. Focusing light generated from plane-wave-illuminated 2D dielectric microcylinders as a function of **(a)** refractive indices and **(b)** particle radii.

2. Energy flow

Classical Mie theory provides an exact electromagnetic solution for light scattering by small particles[2], and shows that PNJs generated from plane-wave-illuminated microparticles can be analysed through the distribution of energy flow, represented by the field lines of time-averaged Poynting vectors around the microparticle[3]. The Poynting vector lines configuration reflects local changes of electromagnetic energy. For 2D full-wave simulations, electric and magnetic vector flux is on the x-z plane. Figures S2a and S2b arrows-denoted Poynting vector, and streamlines-described energy flow in the 2D electromagnetic field corresponding to the cases of the middle part of cylindrical microparticle (MPCM) and length reduced MPCM (R-MPCM) in Fig. 2. Overall, the first light refraction deflects propagating light toward MPCM from the surrounding area, and then diffuses into the external air in the form of divergence after passing through the second light refraction interface (LRI). The focusing spot is inside the microparticle. Aside from the few Poynting vector lines located near the cut sides of relatively low electric intensity areas, the energy flow streamlines around the focal point are parallel with small angular deviations. When MPCM longitudinal length is reduced, as shown in Fig. S2b, the Poynting vector fields focus at the outer edge of the R-MPCM via two convergent light refractions. In this case, the rapid divergence of streamlines away from the focal point originates from the fast convergence of the energy flow streamlines ahead of the focal point, which results in a PNJ width small FWHM beam width.

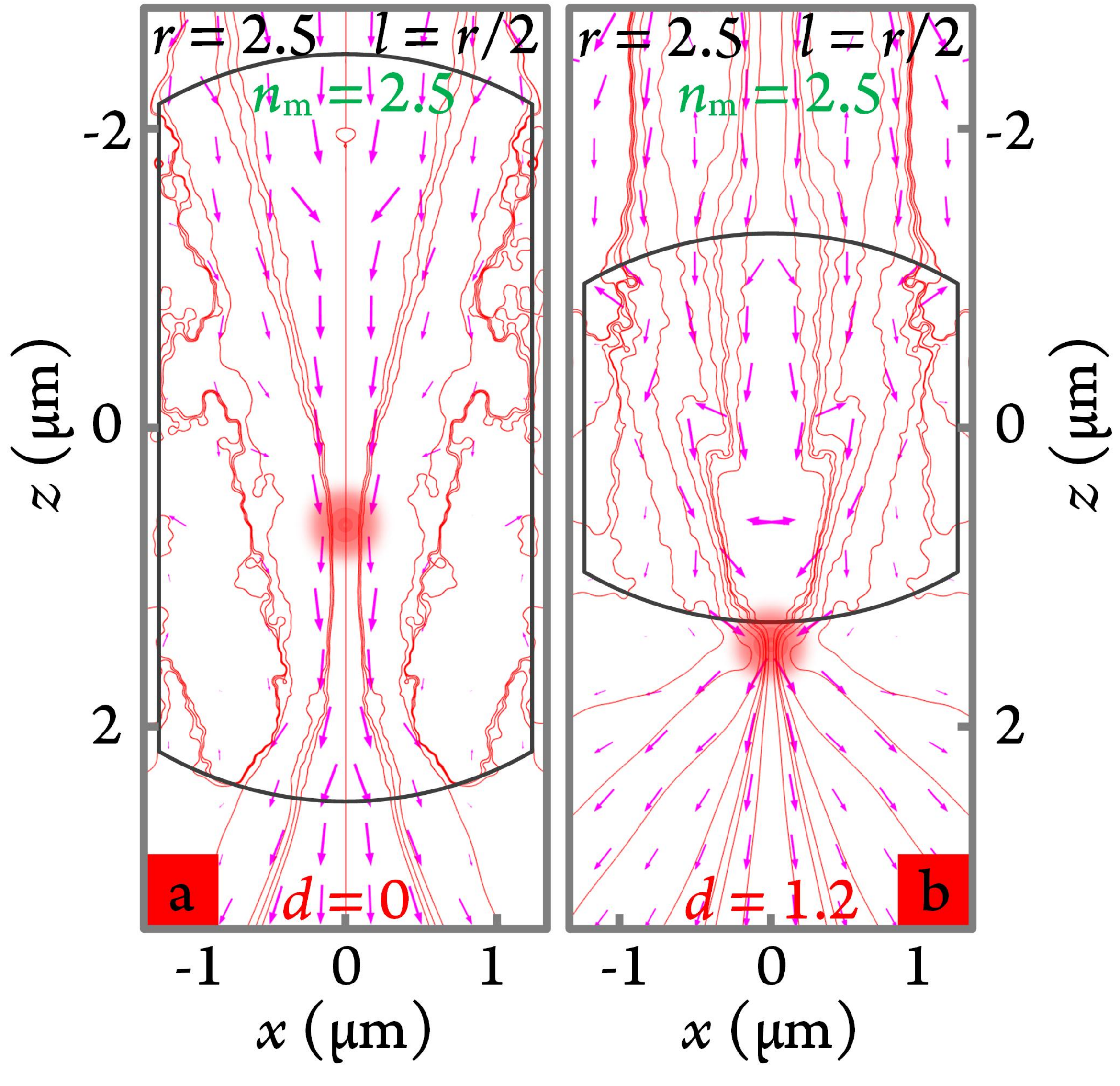

Figure S2. Energy profile for (**a**) MPCM and (**b**) R-MPCM with d = 1.2 μm in the x-z plane. Pink arrows denote the Poynting vector, red lines denote energy flow streamlines. The two red circular spots denote the positions of the focusing light.

3. Electric field distributions

The focusing properties of MPCM and R-MPCM with Ri = 2.25 were numerically explored using FEM method. Figures S3a and S3b show the spatial electric field distributions inside and outside the structures for these two cases, respectively. For drawing convenience, the two field patterns are counter-clockwise rotated ninety degrees and retain their bottom halves containing light focusing parts. The Ri reduction enables a fraction of propagation light to transverse the MPCM and gather in the exterior. However, since most of the light is still focusing in the interior first and then released to the outer space with large divergence angles, the maximum light intensity (I_{max}) is within the MPCM, which can be seen from the lower left panel of normalized intensity ($I_{normalized}$) along the z direction. Even considering the outer gathering optical field, the convergent point of the highest intensity just falls onto the MPCM surface boundary. The FWHM beam width at this position along the direction of z = 2.5 μm is a little less than the classical diffraction limit, but larger than the reported minimum value of 130 nm. For R-MPCM with reduced length d = 1.2 μm, the vast majority of light propagates out and forms a complete long PNJ with I_{max} included. However, the FWHM beam width along the transverse direction of I_{max} (157 nm) also exceeds 130 nm.

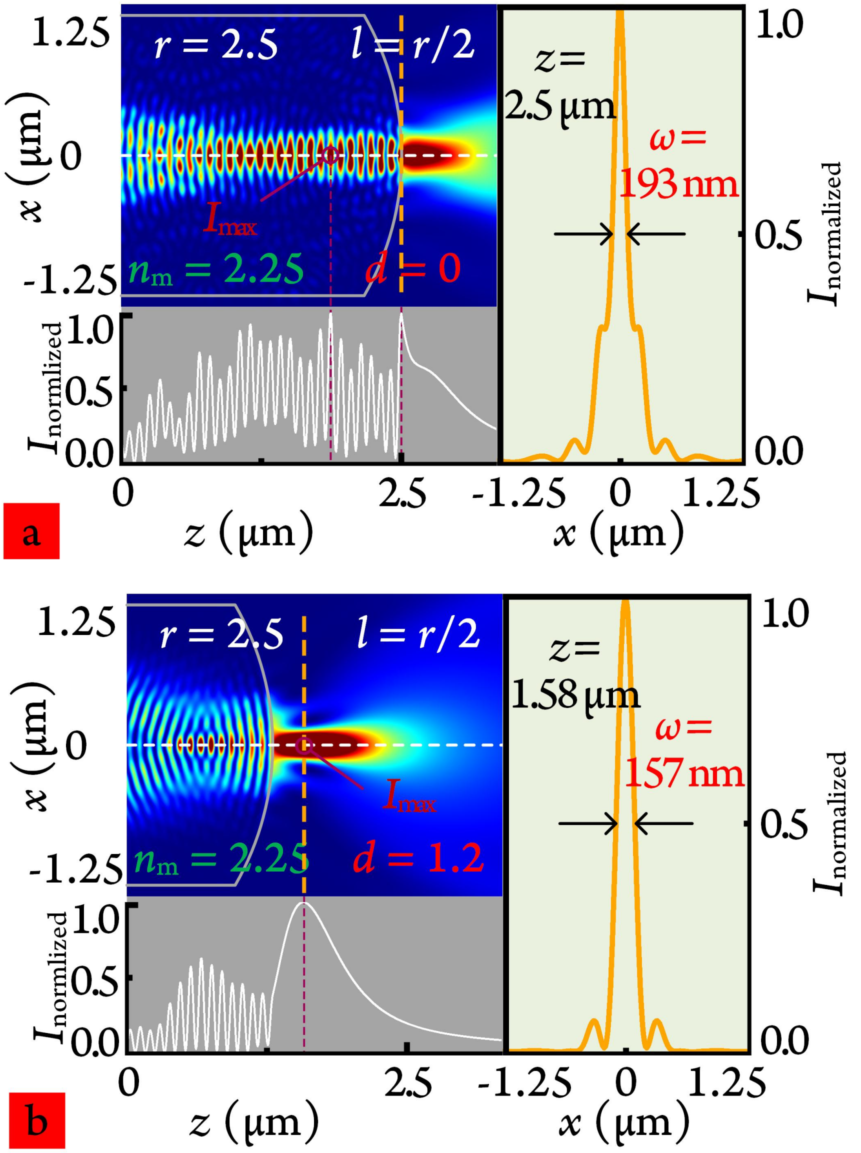

Figure S3. FEM simulation results for (**a**) MPCM with r = 2.5 μm, $l = r/2$, n_m = 2.25 and d = 0; and (**b**) R-MPCM with r = 2.5 μm, $l = r/2$, n_m = 2.25 and d = 1.2 μm. In each case, the upper left-hand panel shows the electric field intensity in the x-z plane; the lower left-hand panel shows the electric field profile along the z axis; and the right-hand panel shows the transverse electric field profile (a) at the tangent edge (z = 2.5 μm) of the MPCM, and (b) at the focal point along x direction.

4. Ray tracing results

The Geometrical optics approximation has provided positive value by allowing analytical calculation of ray-tracing models and qualitative analysis of the variation of light beams transmitting through small particles[4–6]. Figures S4a-S4d show the trajectory results of a plane-wave-illuminated R-MPCMs with four different boundary profiles. Since the *Ri* of the investigated microparticles (*Ri* = 2.5) is larger than the environment air, total internal reflection (TIR) will occur for light propagating from the microparticle into the air. The TIR critical angle is θ_c = arcsin(1/(2.5)) = 23.58°. For the linear shaped (L-type) case, most of the incident light (Fig. S4a, blue lines) will experience two TIRs and one time refraction in the propagation process. The rest are either directly refracted into the air without any TIRs (Fig. S4a, red lines) or refracted out after multiple TIRs (Fig. S4a, green lines). All emergent rays are transmitting parallel to the *z* axis without forming any light focusing.

For the cases of circular shaped (C-type), parabolic shaped (P-type), and harmonic-oscillating shaped (H-type) R-MPCMs, most light rays, with the exception of a small number of beams near the two side edges, successfully pass through the edges and form PNJs at the rear surfaces of the microparticles. Figures S4b-S4d show, the focal point positions and convergence/divergence degree of the emergent rays for P-type R-MPCM are slightly changed relative to C-type R-MPCMs, but significantly changed for H-type R-MPCMs. This is consistent with the different profile boundary curvatures. Such variations of optical field distribution can also be verified by the energy flow lines for C-, P-, and H-type R-MPCMs (as shown in Figs. S4e-S4g).

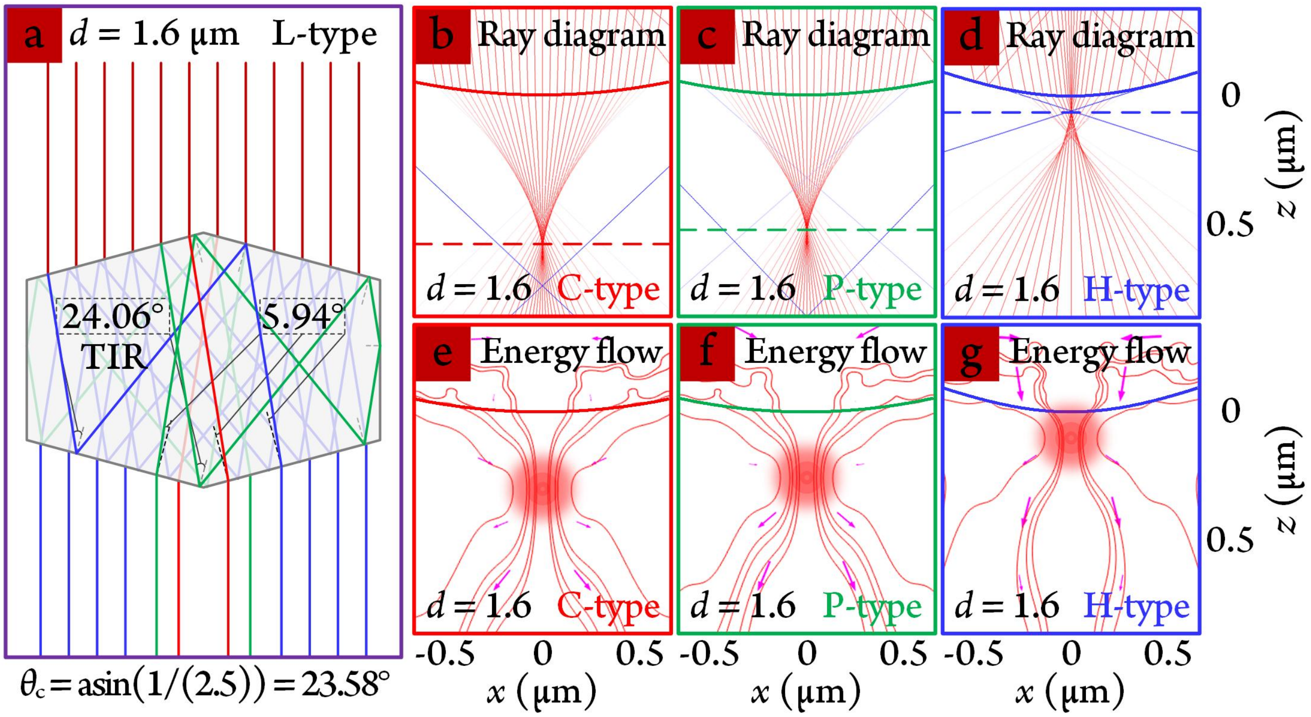

Figure S4. Ray tracing results for a plane wave passing through R-MPCMs (*Ri* = 2.5, *d* = 1.6 μm) with different shape profiles: **(a)** linear-type (L-type), **(b)** circular type (C-type), **(c)** parabolic type (P-type) and **(d)** harmonic-oscillating type (H-type). TIR = total internal reflection, θ_c = TIR critical angle. Corresponding energy flow distributions of **(e)** C-type, **(f)** P-type and **(g)** H-type R-MPCMs. The arrows, lines and spots have the same meanings as in Fig. S2.

References

1. Li, X., Chen, Z., Taflove, A. & Backman, V. Optical analysis of nanoparticles via enhanced backscattering facilitated by 3-d photonic nanojets. *Opt. Express* **13**, 526–533 (2005).

2. Max, B. & Wolf, E. *Principles of optics* (Cambridge University Press, 1999).

3. Wang, Z., Luk'yanchuk, B., Hong, M., Lin, Y. & Chong, T. Energy flow around a small particle investigated by classical mie theory. *Phys. Rev. B* **70**, 035418 (2004).

4. Glassner, A. S. *An introduction to ray tracing* (Elsevier, 1989).

5. Yang, P. & Liou, K. Geometric-optics–integral-equation method for light scattering by nonspherical ice crystals. *Appl. Opt.* **35**, 6568–6584 (1996).

6. Guo, H. *et al.* Near-field focusing of the dielectric microsphere with wavelength scale radius. *Opt. Express* **21**, 2434–2443 (2013).
